# The impact of the introduction of liquid based cytology on the variation in the proportion of inadequate samples between GP practices

**DOI:** 10.1186/1471-2458-7-191

**Published:** 2007-08-01

**Authors:** Wayne N Harrison, Alison MJ Teale, Suzanne P Jones, Mohammed A Mohammed

**Affiliations:** 1 Department of Public Health and Epidemiology, University of Birmingham, Birmingham, UK; 2 West Midlands Specialised Services Agency, Edwin House, Burton on Trent, Staffordshire, UK; 3 Department of Public Health, South Staffordshire PCT, Anglesey House, Towers Business Park, Rugeley, Staffordshire, UK

## Abstract

**Background:**

Historically there has been a wide variation in the proportion of inadequate smears between general practices. Cervical screening in the UK is undergoing a fundamental change by moving from conventional to liquid based cytology (LBC). The main driver for this change has been a predicted reduction in the proportions of inadequate samples. This study investigates the effect of LBC on the variation in the proportion of inadequate samples between general practices using Shewhart's theory of variation and control charts.

**Methods:**

Routinely collected cervical cytology data was obtained for all general practices in two localities in South Staffordshire for periods before and after the introduction of liquid based cytology. Control charts of the proportion of inadequate smears were plotted for the practices stratified by laboratory. A standardised measure of variation for all of the practices in each laboratory and each time period was also calculated.

**Results:**

Following the introduction of liquid based cytology the overall proportion of inadequate samples in the two localities fell from 11.8 to 1.3% (p < 0.05). This fall was associated with a reduction in the average variation between the GP practices in the two localities from 1.6 to 1.0 standard deviations. There has also been a reduction in the number of practices showing special cause variation from eight to one following the introduction of liquid based cytology.

**Conclusion:**

A reduction in the proportion of inadequate samples has been realised in these localities. The reduction in the overall proportion of inadequate samples has also been accompanied by a reduction in variation between GP practices.

## Background

In 2005/06 3.6 million women were screened for cervical cancer in England, 3.36 million following a formal invitation from the screening programme, generating just under 4.0 million cervical smears [1]. Women who have a smear which is identified as inadequate by the reporting cytology laboratory must be retested, in line with national guidelines, and women who have three successive inadequate smears are referred for colposcopy [2]. Inadequate smears are a source of avoidable distress to women, and a potential waste of resources in general practices, clinics and cytology laboratories [2].

A study we undertook in South Staffordshire in 2000–01 identified two sources of variation in the proportion of inadequate smears; that associated with laboratories and that associated with GP practices [3]. Our study showed that there was wide variation in the proportion of inadequate smears amongst 100 general practices, with 23% showing evidence of special cause variation which merited further investigation to identify possible causes. However, the vast majority of general practices (77%) were consistent with common cause variation, which, according to Shewhart's theory of variation, is best addressed by introducing fundamental changes to the underlying process.

Since then a fundamental change has been made in the process of collecting and analysing cervical cytology samples in two of the four localities in the original study, by introducing Liquid Based Cytology (LBC), a new method of cervical cell sample preparation. The main driver for this change was a reduction in the proportion of inadequate samples in pilot studies [4,5].

The current study repeated the analysis carried out previously on this subset of data. The aim is to assess whether the move to LBC has affected the variation in the proportion of inadequate samples between practices as well as the overall proportion.

## Methods

Cervical cytology data were obtained for all 45 general practices in two localities in South Staffordshire for the calendar years 2000 and 2001. The data included the number of samples taken and the number identified as inadequate. The corresponding data for the 45 practices in the two localities was obtained as six month aggregates for October 2005 – September 2006 following introduction of LBC into these areas. Both the laboratories serving the area used the SurePath^® ^LBC system. A number of practice changes have occurred over this period resulting in slightly different numbers using each laboratory in the two study periods.

We undertook analyses of variation by general practice using P-charts. P-charts are one member of the family of control charts and are designed to be used with binomial data (inadequate smear – yes/no) which is expressed as a proportion of the sample size. We sorted our data by the total number of samples (ie sample size) and plotted our P-charts with respect to this order. This has two advantages. Firstly, the resulting P-charts show the impact of sample size on the control limits, and secondly, the resulting control limits are easy on the eye because they appear like a funnel. Such plots have been advocated in health care [6].

A standardised variation in the proportion of inadequate samples was calculated for each practice using the formula:

|Pobs−Pmean|Pmean(1−Pmean)/Nobs
 MathType@MTEF@5@5@+=feaafiart1ev1aaatCvAUfKttLearuWrP9MDH5MBPbIqV92AaeXatLxBI9gBaebbnrfifHhDYfgasaacH8akY=wiFfYdH8Gipec8Eeeu0xXdbba9frFj0=OqFfea0dXdd9vqai=hGuQ8kuc9pgc9s8qqaq=dirpe0xb9q8qiLsFr0=vr0=vr0dc8meaabaqaciaacaGaaeqabaqabeGadaaakeaadaWcaaqaaiabcYha8Hqaaiab=bfaqnaaBaaaleaacqWFVbWBcqWFIbGycqWFZbWCaeqaaOGaeyOeI0Iae8huaa1aaSbaaSqaaiab=1gaTjab=vgaLjab=fgaHjab=5gaUbqabaGccqGG8baFaeaadaGcaaqaaiab=bfaqnaaBaaaleaacqWFTbqBcqWFLbqzcqWFHbqycqWFUbGBaeqaaOGaeiikaGIaeGymaeJaeyOeI0Iae8huaa1aaSbaaSqaaiab=1gaTjab=vgaLjab=fgaHjab=5gaUbqabaGccqGGPaqkcqGGVaWlcqWFobGtdaWgaaWcbaGae83Ba8Mae8NyaiMae83Camhabeaaaeqaaaaaaaa@5420@

Where, for each laboratory in each time period, P_obs _is the proportion of inadequate samples observed for each individual practice, P_mean _the overall mean proportion of inadequate samples for all the practices, and N_obs _the number of samples taken for each individual practice. The mean of the standardised variation for all of the practices in each laboratory and each time period was then calculated. Analyses were performed in Microsoft Excel and Stata.

The study was approved by the Pan South Staffordshire Research Management and Governance Office. The study protocol was also submitted to South Staffordshire Local Research Ethics Committee who ruled that ethics committee approval was not necessary.

## Results

### Overall the proportion of inadequate samples

**Figure 1:**
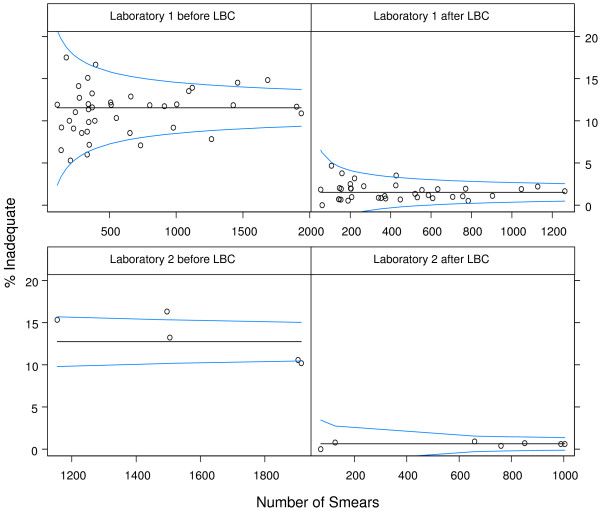
Control charts of the proportion of inadequate samples across GP practices by Laboratory, before (Jan 2000 – December 2001) and after (October 2005 – September 2006) the introduction of LBC. Circles indicate the proportion of inadequate samples for each practice and the blue lines are the upper and lower control limits. Circles between the control limits indicate practices are displaying common cause variation. Those outside either line are displaying special cause variation. Common cause variation is expected variation attributable to "chance". It is part of every process and affects everyone in that process. To reduce common cause variation we need to fundamentally change the underlying process. In contrast, special cause variation is exceptional variation not attributable to "chance", but arising from special circumstances and therefore not affecting everyone in that process.

The overall proportion of inadequate samples for conventional cytology in the two localities for 2000–01 was 11.8% (11.5% for Laboratory 1 and 12.7% for Laboratory 2) (Figure 1). Following the introduction of LBC the overall proportion of inadequate samples fell to 1.3% (p < 0.05) (1.5% for Laboratory 1 and 0.6% for Laboratory 2). There was a slight increase in the proportion of inadequate samples between the first and the second six month of the study period; 1.4% to 1.8% for Laboratory 1 (p = 0.06) and 0.5% to 0.8% for Laboratory 2 (p = 0.21). The ratio in the proportion of inadequate samples after/before the introduction of LBC is 0.11 (95% CI 0.10–0.12) overall and 0.13 (0.12–0.15) for Laboratory 1 and 0.05 (0.03–0.07) for Laboratory 2.

### Variation between GP practices

There has been a reduction in the average variation between the GP practices in the two localities from 1.6 to 1.0 SD in the study period. This has reduced from 1.49 to 1.10 SD for the practices served by Laboratory 1 and from 2.70 to.0.45 SD for the practices served by Laboratory 2.

There has also been a reduction in the number of practices showing special cause variation from eight in the period when conventional cytology was employed to one following the introduction of LBC. None of the practices showing special cause variation in the original study show special cause variation in the current analysis.

## Discussion

The expected reduction in the proportion of inadequate samples following the introduction of LBC has initially been realised in these localities. A systematic review commissioned before the decision (by the National Screening Committee) to roll out LBC found an overall proportion of inadequate samples of 0.8% (95% CI 0.1% to 5.5%), which is comparable to the 1.3% in this study (95% CI 1.2% to 1.5%) [7].

Our findings are contrary to a recent systematic review of primary studies comparing conventional and LBC techniques that found a median difference in the percentage of unsatisfactory slides of only 0.17% (IQR 0.98% to -0.37%) [8]. This difference seems mainly due to a higher proportion of inadequate samples for conventional cytology in practice compared to the primary studies [7]; it remains to be seen if the low proportion of inadequate samples for LBC can be maintained over the longer term.

The introduction of LBC in these two localities has also been associated with a reduction in the variation in the proportion of inadequate samples between GP practices. Because of the relatively low number of samples taken after the introduction of LBC, some practices with zero inadequate samples can still fall above the lower control limit. However, this affects only one practice served by each laboratory so any effect would be minimal. There is further evidence that the introduction of LBC has resulted in a reduction in variation with only one practice now displaying special cause variation compared to the eight practices previously.

It seems therefore that the practices in these two localities are now displaying a more consistent process. This is perhaps not surprising as the introduction of the new technique was accompanied by training for all primary care staff involved in the process as part of local clinical governance arrangements. Training was carried in a centralised fashion by the PCT Cervical Screening Co-ordinators using educational material provided by SurePath^®^. Attendance at a training session was mandatory prior to any primary care practitioner (GP or nurse) undertaking cervical sample-taking with LBC.

Our original study noted a wide variation in the proportion of inadequate samples between all 156 English laboratories [3]. More recently a study has compared the variability of inadequacy rates in eleven Welsh laboratories following the introduction of LBC using longitudinal control charts [9]. They reported a reduction in variability between the laboratories. The results that we present here indicate this reduction may be mirrored at a practice level, suggesting that an improvement has been made in the overall process.

There are a number of limitations in our study. As it is a before/after comparison changes in other factors, such as the demographics of the population screened may have contributed to the results observed. We have demonstrated a large absolute and relative drop in the proportion of inadequate samples, both overall and for each laboratory. However, due to limitations in the data we were unable to adjust this by practice or by age. Nevertheless, the size of the change suggests that confounding factors are unlikely to explain it entirely. We are also unable to estimate how much of the reduction in both the proportion of inadequate samples and the variation between practices is due to LBC as a technique or due to other factors such as mandatory training.

The proportion of inadequate samples are not the only factor to consider when measuring quality improvement in cervical screening. Diagnostic accuracy is also important. In our original study we found no relationship between the proportion of smears reported as inadequate and the positive predictive value of a smear in data from English laboratories [3]. We have not had the opportunity to measure any change in the detection of abnormalities in the current data, though recent reports from two large medium quality studies (based on the criteria used by Davey et al [8]) have indicated that LBC has been associated with an increase in diagnostic accuracy [10,11].

## Conclusion

It is clear that the reduction in the proportion of cervical screening inadequate samples has been realised, at least initially, in these localities. The reduction in the overall proportion of inadequate samples has also been accompanied by a reduction in variation between GP practices.

The challenge now is to maintain the low proportion of inadequate samples in the longer term.

## Competing interests

The author(s) declare that they have no competing interests.

## Authors' contributions

WNH conceived the study, carried out the initial analysis and drafted the paper. AMJT and SPJ obtained the data and revised the draft text. MAM carried out further analysis and revised the draft text. All authors contributed to the final manuscript

## Pre-publication history

The pre-publication history for this paper can be accessed here:


